# Early onset cerebral amyloid angiopathy following childhood exposure to cadaveric dura

**DOI:** 10.1002/ana.25407

**Published:** 2019-01-17

**Authors:** Gargi Banerjee, Matthew E. Adams, Zane Jaunmuktane, G. Alistair Lammie, Ben Turner, Mushtaq Wani, Inder M. S. Sawhney, Henry Houlden, Simon Mead, Sebastian Brandner, David J. Werring

**Affiliations:** ^1^ Stroke Research Centre, Department of Brain Repair and Rehabilitation University College London Queen Square Institute of Neurology and National Hospital for Neurology and Neurosurgery London; ^2^ Lysholm Department of Neuroradiology National Hospital for Neurology and Neurosurgery London; ^3^ Department of Molecular Neuroscience University College London Queen Square Institute of Neurology London; ^4^ Division of Neuropathology National Hospital for Neurology and Neurosurgery London; ^5^ Department of Pathology Cardiff University Cardiff; ^6^ Barts and London School of Medicine and Dentistry Queen Mary University of London and Royal London Hospital London; ^7^ Morriston Hospital Abertawe Bro Morgannwg University Health Board Swansea; ^8^ Medical Research Council Prion Unit at University College London University College London Institute of Prion Diseases London; ^9^ National Prion Clinic National Hospital for Neurology and Neurosurgery London; ^10^ Department of Neurodegenerative Disease University College London Queen Square Institute of Neurology London United Kingdom

## Abstract

Amyloid‐β transmission has been described in patients both with and without iatrogenic Creutzfeldt–Jakob disease; however, there is little information regarding the clinical impact of this acquired amyloid‐β pathology during life. Here, for the first time, we describe in detail the clinical and neuroimaging findings in 3 patients with early onset symptomatic amyloid‐β cerebral amyloid angiopathy following childhood exposure to cadaveric dura (by neurosurgical grafting in 2 patients and tumor embolization in a third). Our observations provide further in vivo evidence that cerebral amyloid angiopathy might be caused by transmission of amyloid‐β seeds (prions) present in cadaveric dura and have diagnostic relevance for younger patients presenting with suspected cerebral amyloid angiopathy. Ann Neurol 2019; 1–7 **ANN NEUROL 2019;85:284–290.**

It is hypothesized that amyloid‐β (Aβ), a hallmark of Alzheimer disease and cerebral amyloid angiopathy (CAA), is transmissible by a similar mechanism to acquired prion diseases.^1^ Initially described in iatrogenic Creutzfeldt–Jakob disease (iCJD),^1^, ^2^, ^3^, ^4^, ^5^, ^6^, ^7^ probable Aβ transmission in the absence of iCJD has been reported following administration of cadaveric human growth hormone,^3^ childhood neurosurgery,^8^ and cadaveric dural graft insertion.^9^ However, the clinical and neuroimaging findings, latency, and range of potential exposure mechanisms for Aβ transmission remain uncertain.

We describe 3 patients diagnosed with symptomatic early onset CAA in a specialist intracerebral hemorrhage service, all with previous childhood neurosurgical or neurovascular procedures using cadaveric dura. Of 663 individual patients referred since January 1, 2015, the 3 described (0.5%) are the only ones with sporadic Aβ‐CAA diagnosed at younger than 50 years. Informed consent was obtained from each patient.

## Case 1

**Figure 1:**
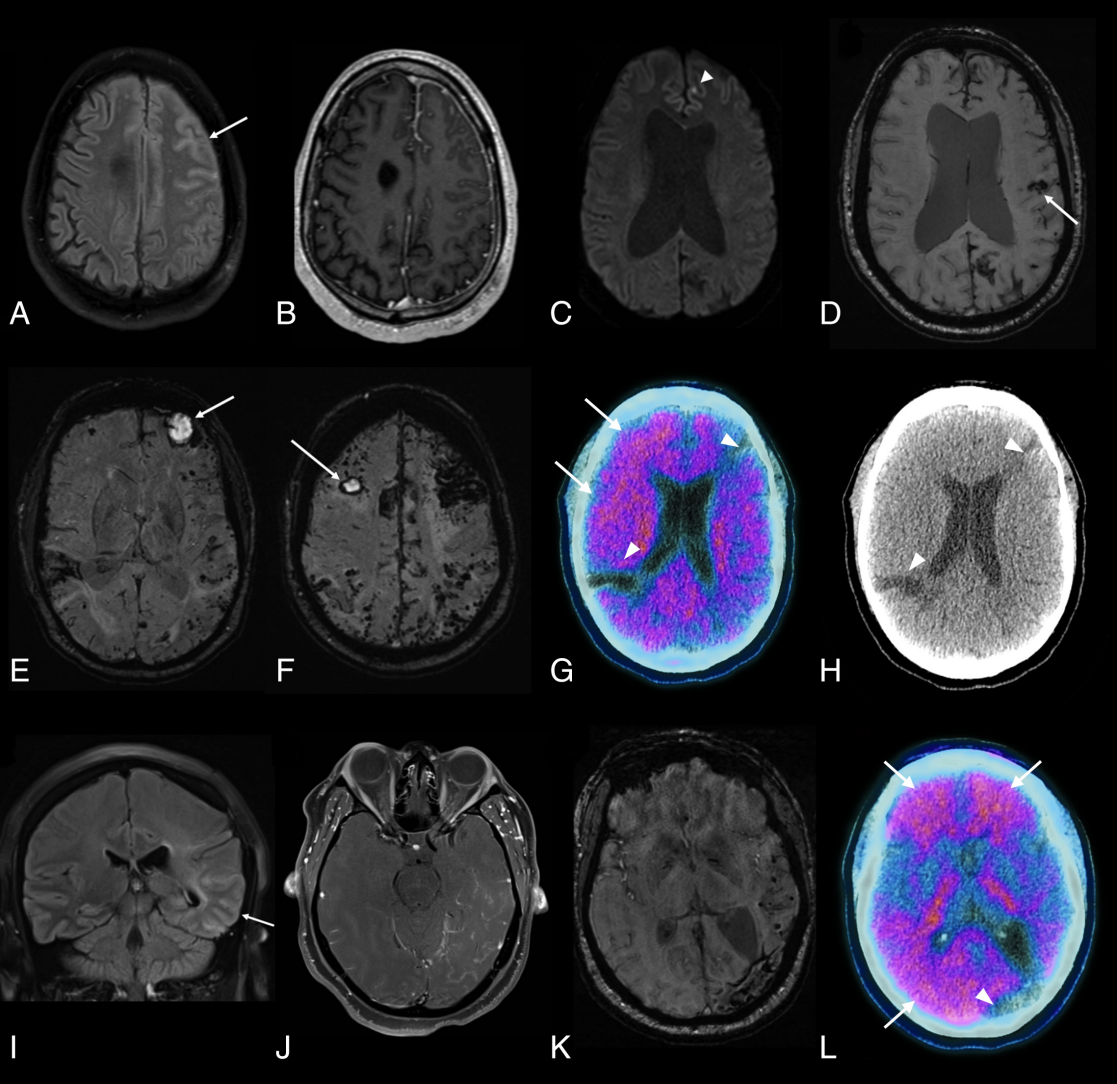
Brain imaging findings. (A–D) Case 1. Fluid‐attenuated inversion recovery (FLAIR; A), T1‐weighted postgadolinium (B), and diffusion‐weighted imaging (C) axial images acquired 2 months after initial presentation demonstrate left frontal gyral swelling and abnormal sulcal signal (*arrow* in A) with leptomeningeal enhancement (B) and a punctate focus of restricted diffusion *(arrowhead)*. (D) Susceptibility‐weighted imaging 4 months later shows a lobar hemorrhage *(arrow)*, multifocal superficial siderosis, and multiple microbleeds. The ventriculomegaly observed in C and D is longstanding (since the choroid plexus papilloma). (E–H) Case 2. (E, F) Susceptibility‐weighted imaging shows hematomas in both frontal lobes *(arrows)* and widespread superficial siderosis and microbleeds, visible as a band of low signal following the leptomeningeal surface of the brain and peripheral, punctate foci of low signal, respectively. (G) ^18^F‐Florbetapir positron emission tomographic (PET)–computed tomographic (CT) imaging shows increased uptake in frontal and parietal cortex (standardized uptake value [SUV] range = 0.0–2.8), suggestive of amyloid deposition *(arrows)*. The 2 areas without uptake *(arrowheads)* correspond with areas of old hemorrhage, as seen on axial CT (H). (I–L) Case 3. FLAIR coronal (I) and T1‐weighted postgadolinium axial (J) images 15 months after initial presentation showing abnormal gyral and sulcal signal in the left temporal lobe *(arrow)* with associated leptomeningeal enhancement. (K) Susceptibility‐weighted imaging 5 months later showing hemosiderin deposition due to previous lobar hemorrhage and numerous temporal microbleeds. (L) Merged ^18^F‐florbetapir PET‐CT imaging shows increased uptake (SUV range = 0.0–3.4) in frontal and posterior (parietal and occipital) cortical regions *(arrows)*; the area with reduced uptake *(arrowhead)* corresponds to the area of old hemorrhagic damage. [Color figure can be viewed at www.annalsofneurology.org]

**Figure 2:**
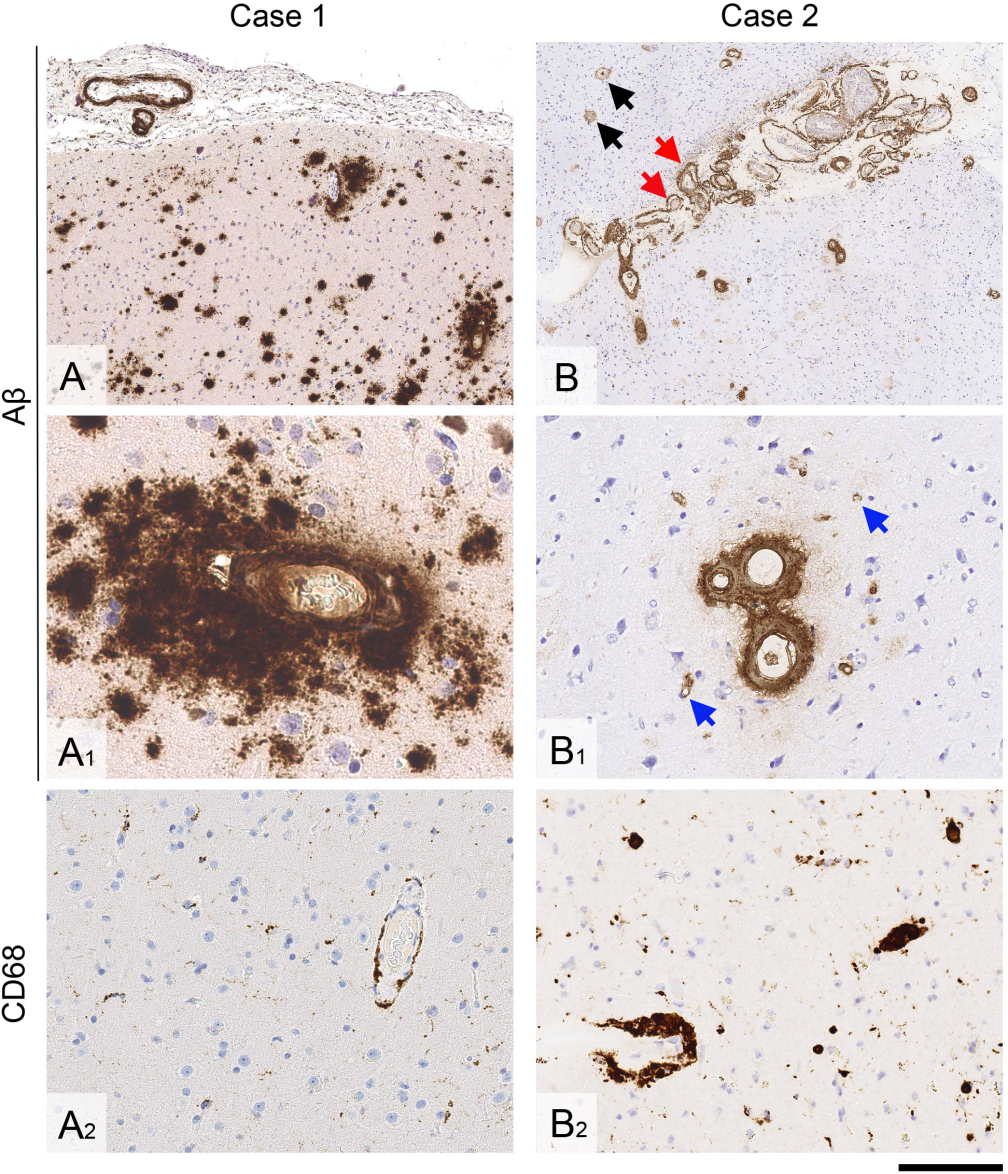
Brain biopsy findings. (A–A2) In Case 1, there is leptomeningeal and cortical cerebral amyloid angiopathy, and widespread diffuse parenchymal amyloid‐β (Aβ) deposits (A and A1). There is only minimal parenchymal and perivascular microglial and macrophage activity (A2). (B–B2) In Case 2, there is particularly widespread leptomeningeal and cortical amyloid angiopathy, including vessel wall splitting (B, *red arrows*) and capillary amyloid angiopathy in the cortex (B1, *blue arrows*). In the cortex, there are also diffuse parenchymal Aβ deposits and rare plaques with central amyloid cores (B, *black arrows*). There are abundant perivascular macrophages, some laden with hemosiderin (B2). Sections A, A1, B, and B1 are immunostained for Aβ; A2 and B2 are immunostained for CD68. All sections are counterstained with hematoxylin. Scale bar = 500 μm in A and B; 50 μm in A1; 100 μm in B1, and 200 μm in A2 and B2. [Color figure can be viewed at www.annalsofneurology.org]

A 48‐year‐old man had 2 generalized tonic–clonic seizures within 1 month. At age 11 years, a choroid plexus papilloma was treated by posterior fossa resection and a cadaveric dural patch (1980) but no radiotherapy. There was no family history of brain hemorrhage or cognitive impairment. Clinical examination revealed longstanding right arm mild pyramidal weakness and ataxia, and slightly unsteady gait. Brain magnetic resonance imaging (MRI) showed patchy T2 hyperintensities bilaterally throughout the cerebral white matter, and 5 punctate foci of restricted diffusion at the gray–white matter interface. Electroencephalography demonstrated intermittent left anterior centrotemporal theta/delta activity enhanced by drowsiness and hyperventilation, with occasional sharp slow waves; he commenced levetiracetam. Carotid duplex, craniocervical computed tomography (CT)‐angiography, bubble‐contrast echocardiography, and 24‐hour electrocardiogram were normal. Two months later, he developed confusion, disorientation, and verbal slowing; brain MRI (Fig 1A–C) showed multifocal abnormal cortical signal and swelling (with adjacent sulcal high signal) on T2‐weighted sequences, most conspicuously in the left frontal region (where there was associated leptomeningeal enhancement and recent subarachnoid hemorrhage) and several new punctate foci of restricted diffusion. Gradient‐recalled T2*‐weighted sequences showed left parietal superficial siderosis and several peripheral microbleeds. A lumbar puncture (performed prior to any clinically manifest intracerebral hemorrhage) showed 680 red blood cells and elevated protein (0.99 g/l). The patient received intravenous methylprednisolone 500 mg daily for 5 days followed by oral prednisolone (50 mg) for a presumed diagnosis of primary central nervous system vasculitis, without clinical response. Three months later, the patient had an acute left frontal intracerebral hemorrhage causing sudden aphasia. Brain biopsy demonstrated leptomeningeal and cortical CAA with scattered leptomeningeal hemosiderin deposits and widespread diffuse parenchymal Aβ deposits, but no perivascular inflammation, vasculitis, or fibrinoid degeneration (Fig 2A, A1, A2); there was no tau pathology or abnormal prion protein (PrP) deposition. *APP*, *PSEN1*, and *PSEN2* gene testing was negative for mutations causing Alzheimer disease or CAA. *APOE* genotype was ε3/ε3. Follow‐up MRI 6 months later showed partial resolution of the left frontal swelling and meningeal enhancement, new parenchymal hemorrhage in the posterior left inferior frontal gyrus, multifocal superficial siderosis, and numerous strictly lobar cerebral microbleeds (see Fig 1D).

## Case 2

A 39‐year‐old man presented with multiple intracerebral hemorrhages. His past medical history included partial resection of a left parotid cavernous hemangioma at age 2 years followed by external carotid embolization using lyophilized cadaveric dura and “gelfoam” emboli (1981). A procedural ischemic stroke, likely secondary to internal carotid embolism, caused right arm weakness. At age 6 years, the patient had further embolization and parotidectomy. He remained well until age 27 years, when he had 3 generalized tonic–clonic seizures associated with a left frontal intracerebral hemorrhage. After treatment with carbamazepine, he remained seizure‐free for 4 years but then had a left frontal lobe intracerebral hemorrhage causing disorientation. He subsequently experienced persistent memory impairment and intermittent confusion with further intracerebral hemorrhages at age 33 years (right parietal) and 35 years (left occipital and right frontal). There was no relevant previous medical or family history. Clinical examination revealed longstanding right hemiatrophy and mild right arm pyramidal weakness. Brain MRI revealed chronic and recent lobar hematomas, patchy superficial siderosis, and innumerable lobar microbleeds (see Fig 1E, F). ^18^F‐Florbetapir amyloid positron emission tomography (PET) showed widespread moderate cortical amyloid deposition (see Fig 1G, H). Cerebrospinal fluid (CSF) examination, performed>1 year after the last symptomatic intracerebral hemorrhage (although contemporaneous MRI showed evidence of clinically silent macrohemorrhage), showed low Aβ1‐42 (261 pg/ml, normal range = 627–1,322 pg/ml) and normal total tau, tau/Aβ ratio, 14‐3‐3 protein, and S100β. Next generation sequencing for mutations in genes associated with dementia (including *APP*, *CHMP2B*, *CSF1R*, *FUS*, *GRN*, *HTRA1*, *ITM2B*, *MAPT*, *NOTCH3*, *PRNP*, *PSEN1*, *PSEN2*, *TARDBP*, *TREM2*, *TYROBP*, and *VCP)* 10 was negative. *APOE* genotype was ε2/ε3. After 2 further intracerebral hemorrhages, brain biopsy confirmed widespread severe leptomeningeal and cortical CAA with parenchymal capillary CAA (see Fig 2B, B1), several cortical microvascular lesions, superficial cortical siderosis, and perivascular hemosiderin‐laden macrophages (see Fig 2B2) but no perivascular lymphocytic inflammation. There were moderate diffuse parenchymal Aβ deposits and occasional core plaques but no tau pathology or abnormal PrP deposition.

## Case 3

A 34‐year‐old woman presented with severe generalized headache and right‐sided visual field loss. Head CT showed acute left parieto‐occipital intracerebral hemorrhage. Two months later she developed generalized tonic–clonic seizures, treated with levetiracetam. Examination revealed mild ideomotor apraxia only. The patient had a significant head injury causing a left parietal skull fracture at age 4 weeks; post‐traumatic focal epilepsy was treated with phenobarbitone and later carbamazepine. At age 3 months, a growing fracture was treated by left parietal craniectomy and cadaveric dural repair (1982).

Brain MRI performed a few weeks after her intracerebral hemorrhage showed several left temporal lobar microbleeds. Digital subtraction angiography (DSA) revealed subtle nonspecific vascular abnormalities around the craniectomy. Imaging 3 months later showed an acute left superior parietal hemorrhage. Repeat DSA showed no new vascular abnormalities. MRI performed 15 months later showed regression of the left temporal hematoma, and mild gyral swelling in the temporal and parietal parenchyma with abnormal sulcal fluid‐attenuated inversion recovery signal, local leptomeningeal enhancement, and additional microbleeds (see Fig 1I–K). ^18^F‐Florbetapir amyloid PET demonstrated widespread cortical amyloid deposition (see Fig 1L). CSF analyses (performed >1 year after the patient's symptomatic hemorrhage) showed low Aβ1‐42 (251 pg/ml, normal range = 627–1,322 pg/ml), low total tau (81 pg/ml, normal range = 146–595 pg/ml) and phospho‐tau (13 pg/ml, normal range = 24 to 68 pg/ml), but normal 14‐3‐3 and S100β. Next generation sequencing for mutations in genes associated with dementia (including *APP*, *CHMP2B*, *CSF1R*, *FUS*, *GRN*, *HTRA1*, *ITM2B*, *MAPT*, *NOTCH3*, *PRNP*, *PSEN1*, *PSEN2*, *TARDBP*, *TREM2*, *TYROBP*, and *VCP)* 10 was negative. *APOE* genotype was ε3/ε3. Repeat MRI, performed 20 months after initial presentation, showed further accumulation of microbleeds.

## Discussion

Our report describes early onset CAA occurring decades after childhood neurosurgical or vascular procedures involving cadaveric dural material, providing further evidence that Aβ might be transmissible as a prion. Furthermore, we provide new evidence that Aβ seeds might also be transmitted via the intravascular route. Finally, in our patients the diagnosis of “iatrogenic” early onset CAA was made in vivo, and, in 2 cases, initially without brain biopsy, on the basis of brain MRI, CSF, and amyloid PET findings.

The characteristic clinicoradiological presentation should help guide noninvasive diagnosis in at‐risk individuals; all patients had prominent recurrent generalized tonic–clonic seizures at or shortly after presentation, and 2 of 3 had abnormal cortical and sulcal signal change—both atypical features in sporadic late onset CAA. This latter imaging finding is similar to changes observed in patients with the inflammatory variant of CAA (CAA‐related inflammation),^11^, ^12^ which is thought to occur as a consequence of spontaneously generated anti‐Aβ autoantibodies^13^ and bears a close resemblance to the amyloid‐related imaging abnormalities (ARIA) that can occur after treatment with anti‐Aβ immunotherapy.^14^ Both ARIA and CAA‐related inflammation can be either mildly symptomatic or asymptomatic,^15^, ^16^, ^17^ leading to speculation that this might represent physiological attempts at Aβ clearance.^18^ Although we cannot confirm that this mechanism is relevant in our patients, it raises the interesting possibility that Aβ from exogenous dural material might be recognized as nonautologous and thus provokes a more marked immune response.

In keeping with the few previous reports,^8^, ^9^ our patients first developed neurological symptoms approximately 3 to 4 decades after exposure to cadaveric dura (range = 27–37 years), suggesting a long incubation period. The association between CAA and endovascular embolization with cadaveric dura suggests that Aβ seeds might be transmitted without direct neurosurgical brain exposure. Lyophilized cadaveric dura embolization material has been suggested to potentially transmit iCJD,^19^, ^20^ and experimental intravenous injection of Aβ seeds causes CAA.^21^ In 2 of our cases (Cases 1 and 2), there was evidence of significant amyloid angiopathy distant from the site of presumed transmission; this is in keeping with previously reported cases of suspected iatrogenic CAA, where widespread disease is observed on neuropathological examination.^8^, ^9^ There are 2 potential explanations for these observations. First, although prions do spread along defined neuroanatomical pathways, there is evidence that they can reach specific target regions despite different initial sites of inoculation^22^; in the case of Aβ, the target might be the cerebral and leptomeningeal vasculature, resulting in widespread disease of these vessels regardless of transmission method. Second, prions are believed to propagate in 2 stages, an exponential replication phase and a subsequent plateau phase where prion concentration is maximal.^22^ It is during this latter period that both clinical symptoms and visible neuropathology become manifest, and it has been hypothesized that this represents a “selective cellular vulnerability” to the high prion burden^22^; if cerebral and leptomeningeal blood vessels are particularly susceptible to Aβ burden in this way, this again could result in the widespread CAA observed.

In the United Kingdom, human dura mater grafts were banned in 1992; information on patients receiving dura grafts before this ban is not available,^23^ making it difficult for us to estimate the risk of transmission. Although iatrogenic CJD following dural transplantation was associated with a single graft brand (Lyodura), it is not clear whether this will also be the case for Aβ; the situation is further complicated as, compared to PrP, Aβ is relatively common. Although the number of dural grafts performed in the United Kingdom is unknown, there are data from other countries. In Japan, about 20,000 patents received Lyodura grafts annually from 1983 to 1987, whereas in the USA, approximately 4,000 patients received dural transplants per year, of which <10% were Lyodura.^24^ In Australia, 1,172 patients were exposed to Lyodura between 1982 and 1986.^25^ It is worth noting that lyophilized cadaveric dura has also previously been used for non‐neurosurgical (for example, head and neck, cardiothoracic, abdominal, and urological^19^, ^20^, ^25^) procedures.

We acknowledge important limitations. We lack comprehensive information about the transplanted dura (for example, batch or manufacturer details), so cannot establish causality, or prove that transplanted dura contained Aβ seeds. We cannot reliably estimate the population exposed to cadaveric dura potentially containing Aβ seeds, because information on patients receiving a dura mater graft in the United Kingdom is not available, as noted above. We lack pathological proof that the patient described in Case 3 does not have iCJD, but the imaging findings (with 90% sensitivity^26^), negative CSF 14‐3‐3 (with 80% specificity^27^), and absence of relevant symptoms strongly argue against this diagnosis. We were also unable to test for amyloid precursor protein (APP) duplications in our patients, which are associated with early onset Alzheimer disease together with CAA.^28^, ^29^ However, patients with APP duplications described to date have presented with progressive cognitive impairment, making this an unlikely cause of the clinical syndromes of the patients described here, who all had initial symptoms related to intracerebral hemorrhages or seizures; additionally, there is no evidence of a family history suggestive of APP duplication for any of the cases described.^28^, ^29^, ^30^, ^31^, ^32^, ^33^

We also acknowledge that our cases might be prone to ascertainment bias and are unlikely to represent the full spectrum of iatrogenic CAA. Our cases prompted further intensive investigation because of their relatively young age at presentation; patients transplanted before the mid‐1970s or those transplanted after this time but at an older age (as adults) would have presented at a typical age for sporadic CAA (ie, >55 years), and thus are unlikely to have been investigated in the same detail. In the United Kingdom, iCJD was described following dural transplantation as early as 1969.^34^ However, 80% of cases of iatrogenic CJD identified worldwide were exposed between 1983 and 1987^34^; whether this is relevant to the narrow window of dural Aβ exposure seen in the patients described here (1980–1982) is unknown. We cannot rule out the possibility of exposure of our patients to a “point source”; 2 of our cases (Cases 1 and 3) were treated by the same operating surgeon at the same center, so it is conceivable that samples came from a single donor or batch. However, Lyodura contains dura from different donors^34^, ^35^ so this common link between patients could simply reflect the surgeon's brand preference. With the benefit of hindsight, we now specifically ask all patients presenting with CAA about a prior history of surgical intervention, regardless of age, and we suspect that other iatrogenic cases will come to light as a consequence. The identification of further cases, by our center and others, will be essential for definitively establishing the nature and extent of iatrogenic CAA, something that is beyond the scope of this case series in isolation.

We also recognize that CAA in our patients might have resulted from mechanisms other than dural Aβ transmission. A recent neuropathological case series^8^ describing Aβ transmission following childhood neurosurgery (n = 4) reported that no dura was used in 1 case, and that its use was unlikely (although not completely excluded) in the other 3, so our study cannot exclude transmission via neurosurgical or neurovascular instrumentation. Additionally, it is possible that brain trauma (either external insults affecting the head or that secondary to neurosurgical instrumentation) could impair perivascular clearance pathways for Aβ; indeed, associations between early onset CAA and traumatic brain injury have been described.^8^, ^36^ However, many of these cases also underwent neurosurgical intervention and, in our series, case 2 had no prior history of either direct head trauma or neurosurgical intervention.

Our report increases the number of patients and documented mechanisms of transmission for early onset (“iatrogenic”) CAA, implicating the likely molecular mechanism of transmissible Aβ seeds (prions) in cadaveric dura. The lengthy presymptomatic phase raises the possibility that affected individuals treated with cadaveric dura many years previously could potentially transmit Aβ seeds. We provide new evidence that this transmission can result in symptomatic disease during a patient's lifetime and is not simply an incidental postmortem finding. If confirmed in larger, more definitive epidemiological studies, our conclusions could have implications for public health precautions, to avoid future clinical transmission and ensure safe laboratory practices for handling tissues containing Aβ pathology.

## Author Contributions

G.B. and D.J.W. contributed to the conception and design of the study; all authors contributed to the acquisition and analysis of data; all authors contributed to drafting the text and preparing the figures.

## Potential Conflicts of Interest

Nothing to report.
